# Stretchable Strain Sensor for Human Motion Monitoring Based on an Intertwined-Coil Configuration

**DOI:** 10.3390/nano10101980

**Published:** 2020-10-07

**Authors:** Wei Pan, Wei Xia, Feng-Shuo Jiang, Xiao-Xiong Wang, Zhi-Guang Zhang, Xia-Gui Li, Peng Li, Yong-Chao Jiang, Yun-Ze Long, Gui-Feng Yu

**Affiliations:** 1 College of Chemistry and Pharmaceutical Sciences, Qingdao Agricultural University, Qingdao 266109, China; tcpanwei@126.com (W.P.); xw2508717257@163.com (W.X.); nosaygiveup@126.com (F.-S.J.); 2 Collaborative Innovation Center for Nanomaterials & Devices, College of Physics, Qingdao University, Qingdao 266071, China; wangxiaoxiong69@163.com; 3 College of Science and Information, Qingdao Agricultural University, Qingdao 266109, China; zhangzhiguangphysics@126.com (Z.-G.Z.); qdguixiali@126.com (X.-G.L.); pg.lee@163.com (P.L.); qdycjiang@126.com (Y.-C.J.); 4 State Key Laboratory of Bio-Fibers and Eco-Textiles, Qingdao University, Qingdao 266071, China

**Keywords:** stretchable, strain sensor, intertwined-coil configuration, wearable

## Abstract

Wearable electronics, such as sensors, actuators, and supercapacitors, have attracted broad interest owing to their promising applications. Nevertheless, practical problems involving their sensitivity and stretchability remain as challenges. In this work, efforts were devoted to fabricating a highly stretchable and sensitive strain sensor based on dip-coating of graphene onto an electrospun thermoplastic polyurethane (TPU) nanofibrous membrane, followed by spinning of the TPU/graphene nanomembrane into an intertwined-coil configuration. Owing to the intertwined-coil configuration and the synergy of the two structures (nanoscale fiber gap and microscale twisting of the fiber gap), the conductive strain sensor showed a stretchability of 1100%. The self-inter-locking of the sensor prevents the coils from uncoiling. Thanks to the intertwined-coil configuration, most of the fibers were wrapped into the coils in the configuration, thus avoiding the falling off of graphene. This special configuration also endowed our strain sensor with an ability of recovery under a strain of 400%, which is higher than the stretching limit of knees and elbows in human motion. The strain sensor detected not only subtle movements (such as perceiving a pulse and identifying spoken words), but also large movements (such as recognizing the motion of fingers, wrists, knees, etc.), showing promising application potential to perform as flexible strain sensors.

## 1. Introduction

Flexible conductive wearable devices have drawn great attention for the dramatic development of artificial intelligence technology [1,2,3,4,5,6,7]. Strain sensors, as a fundamental type of flexible conductive wearable electronics, have become a building block of electronic devices. They can monitor the temperature and humidity, be sensitive to light source power deviations, and they can detect ultraviolet (UV) light and volatile organic compounds (VOCs). The sensors can also recognize the movement of humans, including subtle movements (such as perceiving a pulse and identifying spoken words) and large movements (such as recognizing the motion of fingers, wrists, and knees). Additionally, strain sensors have demonstrated the ability to sense the body’s health parameters (e.g., the quantitative glucose value) [8,9,10,11,12,13,14,15,16,17,18,19,20,21,22]. In addition to the requirements of durability, low fabrication cost, biocompatibility, and light weight, two important considerations that remain are the fulfillment of a high sensitivity and a large strain range simultaneously [23,24,25].

Most flexible strain sensors can only tolerate a very limited strain extent, e.g., usually less than 200% [26]. To date, two major approaches have been employed to improve the stretchability. One is using materials that are stretchable themselves. For example, liquid metals, ionic conductors, and organic conducting polymers have been widely introduced. The other approach is designing a new configuration. For example, because of the small testing areas of fiber-shaped stretchable strain sensors, they have been woven into textiles to achieve wearability and integrality [27,28]. Additionally, wavy configurations, wrinkled configurations, percolating networks, helical structures, a serpentine mesh, and spring-like configurations have been adopted to increase stretchability [29,30,31,32,33,34,35,36,37,38]. Here, it should be noted that a coil structure, a helical-coil structure, and a supercoil structure have also been employed to provide structural stretchability [39,40,41]. Tight twisting of fibers induced strong van der Waals forces, resulting in a significantly enhanced mechanical strength and an enhanced Young’s modulus [42]. Additionally, the stretchability of devices with helical-coil structures was reported to be higher than that of devices with coil structures. For example, coiled fibers which were fabricated by twisting [43] had a stretchability of 400%. In contrast, helical-coil fibers possessed a stretchability of 850%; they were fabricated by winding active fibers around the surface of a nonactive core substrate based on coiled fibers [40,44]. Additionally, the stretchability of the super-coil structures was 1500% [44]. Here, an interwined-coil configuration is designed for the first time, which is a new structure that has not yet been reported. The intertwined-coil configuration not only fulfills the stretchability requirement and improves the recovery, but also prevents the coils from uncoiling.

Conductive nanomaterials play an important role in increasing the sensitivity of strain sensors. For example, conducting polymers, nanowires, metal nanoparticles, carbon nanotubes, and ionic liquids have been deposited on the surface or embedded in the matrix of a strain sensor to improve its sensitivity. Because of its large surface area, high thermal conductivity, good chemical resistance (to acids, bases, and salts), good thermal stability, and unique electrical and mechanical properties, graphene has attracted considerable attention for use in strain sensors [16,23,45,46,47,48,49,50,51,52]. Chemical vapor deposition, vacuum filtration, and combining of graphene with an elastomer have been applied to obtain graphene-based strain sensors. However, achieving high stretchability remains challenging. [9,21,34,35,53] 

Here, a highly stretchable strain sensor based on thermoplastic polyurethane (TPU) and graphene with an intertwined-coil configuration was fabricated. The fabrication process is simple, efficient, and economical. 

## 2. Experimental Section

**Figure 1:**
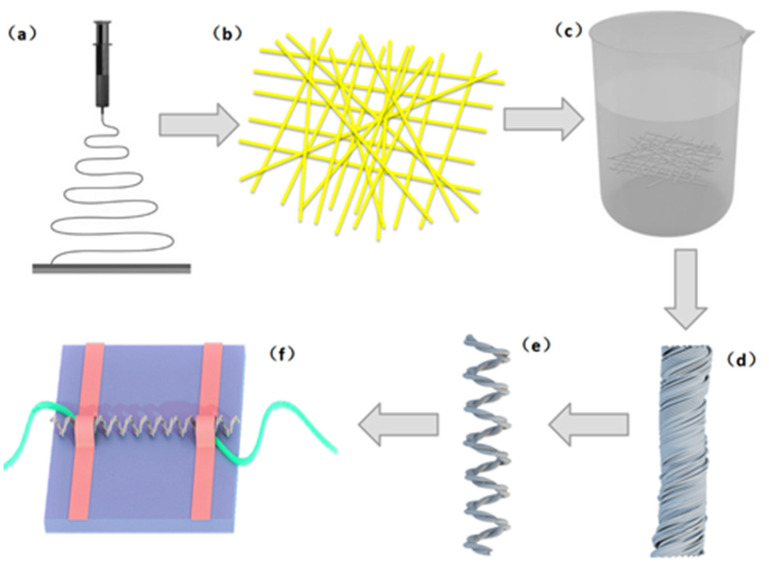
Preparation process of fabricating a thermoplastic polyurethane (TPU)/graphene strain sensor with an intertwined-coil configuration: (**a**,**b**) electrospinning technique; (**c**) dip-coating technique; (**d**,**e**) spinning technique; and (**f**) the properties of strain sensor measurement.

A highly stretchable TPU/graphene strain sensor with an intertwined-coil configuration was prepared through electrospinning, dip-coating, and spinning. A schematic illustration of the fabrication strategy is shown in Figure 1.

### 2.1. Materials

TPU was purchased from Bayer. Graphene was purchased from Zhongsen Linghang Co., Ltd. (Shanghai, China). Acetone and *N,N*-dimethylformamide (DMF) were supplied by Sinopharm Chemical Reagent Co., Ltd (Beijing, China). All materials were used without further purification.

### 2.2. Electrospinning of the TPU Nanomembrane (Schematic a–b)

**Figure 2:**
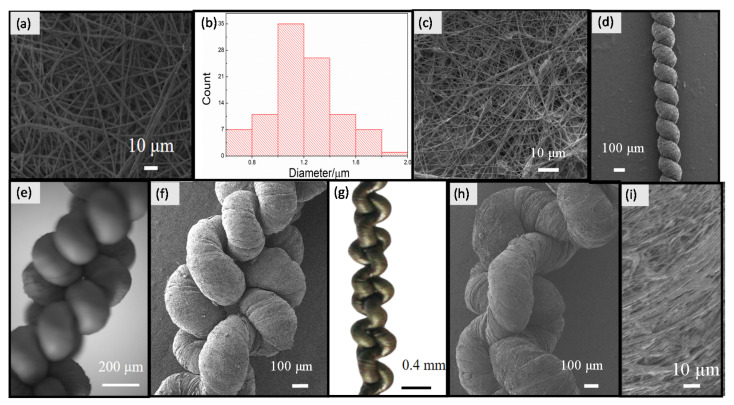
(**a**) Scanning electron microscopy (SEM) image of the TPU nanofibrous membrane; (**b**) diameter distribution of the electrospun TPU membrane; (**c**) SEM image of the TPU/graphene nanofibrous membrane; (**d**) SEM image of the TPU/graphene strain sensor with a spring-like configuration; (**e**) optical microscope image of the TPU/graphene strain sensor with a double spring-like configuration; (**f**) SEM image of the sensor in a double spring-like configuration; (**g**) optical microscope image of the TPU/graphene strain sensor with an intertwined-coil configuration; (**h**) SEM image of the TPU/graphene strain sensor with an intertwined-coil configuration; (**i**) high magnification SEM image of the TPU/graphene strain sensor with an intertwined-coil configuration.

To obtain a uniform TPU electrospun membrane, 2.3 g TPU was dissolved in 7.7 g mixed solvent of acetone and DMF. Then, the mixture was stirred for 4 h at 40 °C until the mixture was clear, viscous, and homogenous. After that, the solution at a concentration of 23 wt% was drawn into a 10 mL plastic syringe, which was connected to a needle with a diameter of 0.4 mm. A high voltage of 13 kV was applied to the tip of the needle. The solution was driven by a syringe pump at a speed controlled at 1 mL·h^−1^, and the distance between the syringe needle and the collector was 12 cm. Finally, a TPU electrospun membrane was collected on a rotating drum covered with aluminum foil for 30 min (Figure 2a,b) [54].

### 2.3. Preparation of the TPU/Graphene Membrane (Schematic c)

A graphene solution was obtained by adding 3 g graphene into 70 g alcohol. Ultrasonic treatments were carried out for 20 min. Then, the solution was stirred for 24 h until a homogeneous dispersion was achieved. After that, the TPU membrane (20 cm length and 10 cm width) was completely immersed into the well-dispersed graphene solution with ultrasonic treatments. Treatment time was increased to 3 min, which allowed more graphene to adhere onto the surfaces of the TPU membrane (Figure S1a). To obtain high mass concentrations of graphene, the dip-coating procedure was repeated six times (Figure S1b,c). Owing to capillary action, the color changed from white to black. Once the solvent had evaporated, graphene solution was deposited onto the surface of the TPU membrane. Finally, the aligned TPU/graphene membrane was washed with deionized water for several times, and dried at 60 °C in an oven for 4 h. Finally, the TPU/graphene membrane (Figure 2c) was dried in an oven.

### 2.4. Preparation of the TPU/Graphene Strain Sensor with an Intertwined-Coil Configuration (Schematic d-e)

The TPU/graphene strain sensor with an interwined-coil configuration was fabricated as follows: first, one end of the TPU/graphene membrane (40 cm in length and 0.3 cm in width) was fixed to a motor, with the other end fixed to an iron that could move. The TPU/graphene membrane was transformed into a yarn (38 cm in length and 0.025 cm in width) with the rotation of the motor at an initial speed of approximately 100 rpm.

Then, spring coils were formed in sequence by adjusting the rotation speed to 60 rpm. The yarn shortened continuously until all the yarn was converted into uniform coils (Figure 2d). Eventually, a sensor with a spring-like configuration was obtained. 

A double-spring configuration was fabricated by folding the sensor with a spring-like configuration in two (Figure 2e,f). The strain sensor with a double-spring configuration was stretched to a degree of 100% and then released. In the process of releasing, the helical coils intertwined with each other. As the double-spring configuration recovered back to its original shape, the helical coils thoroughly intertwined. Thus, an intertwined-coil configuration with even intertwining of the coils was eventually obtained through stretching and releasing processes (Figure 2g,h and Figure S1d,e).

One significant advantage of this fabrication method was that most of the graphene were fixed in the configuration so that the conductive networks were stabilized. Furthermore, the intertwined coils ensured large stretchability and high recovery. Additionally, the simple and low-cost preparation process enabled scaled-up production. Finally, uncoiling was avoided due to the intertwined configuration. 

### 2.5. Electrode Fabrication (Schematic f)

Two copper wires were immobilized with silver paste onto the two ends of the sensor and then were fixed with Cu tape for better contact, to form the electrodes.

### 2.6. Characterizations

The morphology and structure of the strain sensor was characterized by scanning electron microscopy (SEM, Hitachi S4800, Tokyo, Japan). An optical microscope was used to record optical microscope images (Olympus, BX53, Tokyo, Japan). Optical images were obtained through an optical camera (Olympus EM10, Tokyo, Japan). The electrical characteristics were measured by an Agilent 9200 (State of California, America) high resistance meter system. The long time stretching-releasing experiments were carried out using a uniaxial testing machine (ZDW-2HA865, Times Brilliant CO., Ltd, Hangzhou, China) at a tensile rate of 20 mm/min.

## 3. Results and discussion 

### 3.1. Morphology

Figure 2a shows a SEM image of the TPU nanofibrous membrane. It shows that the average diameter of the TPU nanofibers is approximately 1235 nm (Figure 2b). The nanofibers form a dense array, and the thickness of the membrane that was electrospun for 30 min was approximately 0.016 mm. Figure 2c presents a SEM image of the TPU/graphene nanofibrous membrane. Compared to the pure TPU membrane, the TPU/graphene nanofibrous membrane was not homogeneous, due to the existence of graphene. A SEM image of the sensor with a spring-like configuration is shown in Figure 2d, which shows that the TPU/graphene nanofibrous membrane has been twisted into microscale homogeneous coils. Figure 2e presents an optical microscope image of the sensor in a double spring-like configuration, and Figure 2f shows the SEM image. Figure 2g presents an optical image of the sensor in an intertwined-coil configuration, and Figure 2h shows the SEM image. The coils thoroughly intertwined with each other to obtain self-inter locking, which prohibited the coils from relative rotation. It should be noted that a microscale gap existed between the coils. A high magnification SEM image of the sensor in the intertwined-coil configuration is shown in Figure 2i, which shows an interconnected graphene conductive network on the surface of the strain sensor. Additionally, the direction of the fiber is in accordance with the direction of the spinning. Here, it should be noted that the fibers should form nanoscale gaps among the TPU/graphene fibers during the spinning process. Then, the nanoscale gaps among the TPU/graphene fibers and microscale gaps among the coils in this case, simultaneously increased the stretchability of the TPU/graphene strain sensor [43,55]. It should be noted that most of the graphene was rolled inside of the coils. On the one hand, the graphene made contact with other layers, forming a conductive network that enhanced the conductivity of the sensor. On the other hand, most of the graphene remained unchanged and avoided being peeled off in the process of stretching.

### 3.2. Raman Spectrum

**Figure 3:**
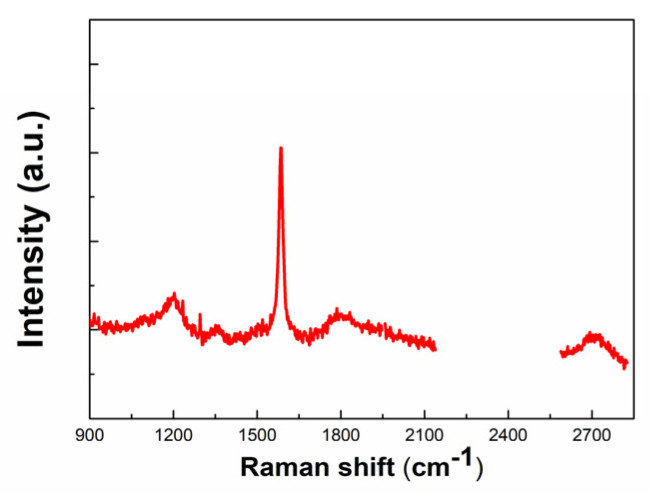
Raman spectrum of the graphene/TPU strain sensor.

The Raman spectrum of the TPU/graphene strain sensor is shown in Figure 3. It exhibits two characteristic peaks of graphene. The D band is located at approximately 1352 cm^−1^, which is attributed to sp^3^ defects. The 2D band is located at 2698 cm^−1^. The G band appears at 1584 cm^−1^, due to the stretching vibration of sp^2^ hybridized carbon atoms [56,57,58,59]. The characteristic peaks of TPU may become weaker and even disappear with the loading of grapheme [56,57]. The weak peak at 880 cm^−1^ is assigned to the stretching vibrations of CH_2_. The peak at 1200 cm^−1^ represents aromatic =C-H in-plane deformation vibrations. The peak at 1770 cm^−1^ is attributed to the free carbonyl group [57,60].

### 3.3. Stretchability and Recovery 

The relationship between the conductivity of the strain sensor and graphene coating cycle was also examined. The conductivity of the strain sensor increased with increasing coating cycle from 0.055 to 2.303 S·cm^−1^ (Figure S2).

**Figure 4:**
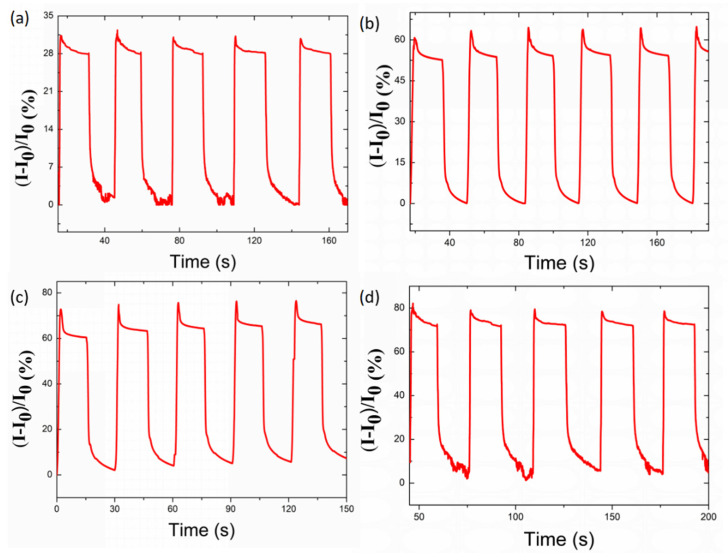
Current change of the TPU/graphene strain sensor with an intertwined-coil configuration subjected to (**a**) 100%, (**b**) 200%, (**c**) 300%, and (**d**) 400% tensile strain and then strain release in each cycle.

The performance of the strain sensor was demonstrated first by investigating the recovery from stretching. Figure 4 shows the response of the strain sensor to stretch–release cyclic elongation at different strain levels (100% strain for Figure 4a, 200% strain for Figure 4b, 300% strain for Figure 4c and 400% strain for Figure 4d). The sensor recovered back to its original electrical and mechanical features, even after being subjected to 400% strain. This value is higher than the key stretching points of human motion. For example, the stretching limit of knees and elbows is up to 50% [50].

**Figure 5:**
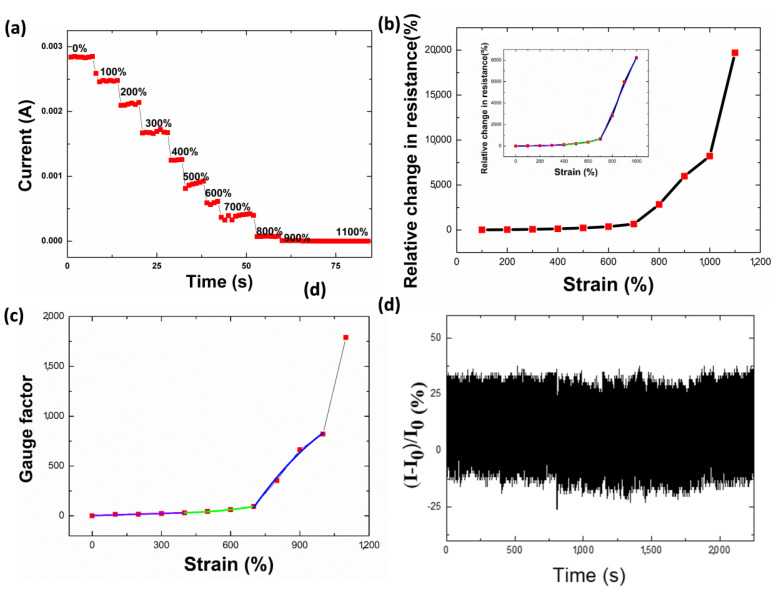
(**a**) I-T characteristic behavior of the TPU/graphene composite strain sensor with an intertwined-coil configuration under different tensile strains. (**b**) Plots of relative resistance change versus strain of the TPU/graphene composite strain sensor with an intertwined-coil configuration in the range of 0% to 1100% strain. The insert is the relative resistance change versus strain of the TPU/graphene composite strain sensor with an intertwined-coil configuration in the range of 0% to 1000% strain. (**c**) Plots of gauge factor versus strain of the TPU/graphene composite strain sensor with an intertwined-coil configuration. (**d**) One thousand cycles of a repeated stretching–releasing test under strain from 0% to 150%.

The intertwined-coil configuration design of the TPU/graphene composite strain sensor also endows it with unique stretchability. To detect the amount of stretching that the sensor with an intertwined-coil configuration can tolerate, the sensor was stretched along the axis with a step of 100% strain. The current characteristic curve of the TPU/graphene composite strain sensor under different strains is depicted in Figure 5a. A remarkable sensitivity to each stretching step is observed, e.g., the current decreases gradually with the variation of the strain. It should be noted that the maximal strain listed in Figure 5a is 1100%, indicating the high stretchability of the strain sensor. The sensor can sustain a high stretchability, owing to the intertwined-coil configuration and the synergy of the two structures (nanoscale fiber gap and microscale twisting of the fiber gap). Additionally, in the process of twisting, a strong van der Waals force can be generated between the nanowires, which can enhance the mechanical strength and Young’s modulus [42,55].

**Figure 6:**
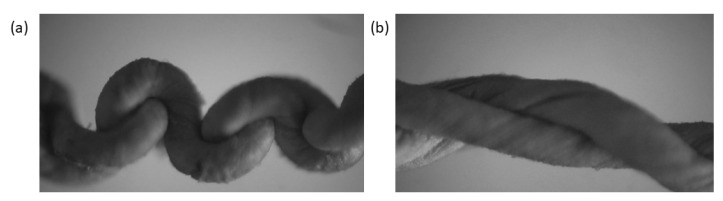
Optical microscope image of the TPU/graphene strain sensor with an intertwined-coil configuration under (**a**) 400% strain and (**b**) 800% strain.

In the process of stretching, the configuration of the sensor undergoes the following process. The first stage is the stage in which the intertwined-coil configuration can be recovered (under 0–400% strain, Figure 6a). In this stage, the stretchable strain sensor recovers to the original state while it is stretched up to a degree of 400%. In this process, large and small coils appeared accompanying the stretching and releasing procedure. The second stage is the steady opening of the coils, and at this point the coils of the intertwined configuration cannot recover to their original configuration (under 400–800% strain, Figure 6b, Figure S1f). The third stage is the occurrence of minor cracks, that is, macroscopic cracks, until the sensor fails (strain larger than 800%). The strain sensor, based on an electrospun TPU nanofibrous membrane and in situ polymerization of polyaniline (PANI), tolerates a strain up to 165% at the point of the sensor rupture [61]. The strain that the electrospun TPU/PVDF composite membrane tolerated was less than 100% [62]. The strain sensor based on reduced graphene oxide (RGO)-decorated TPU electrospun fibrous membranes tolerated a strain up to 660% [45,63].

The relative resistance change is defined as ΔR/R_0_, where ΔR = R-R_0,_ R_0_ is the resistance in the original state, and R is the real-time resistance when the sensor is stretched. The relative resistance change versus strain of the TPU/graphene composite strain sensor with an intertwined-coil configuration in the range of 0% to 1100% strain is shown in Figure 5b. This plot is divided into four stages: 0% to 400%, 400% to 700%, 700% to 1000%, and 1000% to 1100% strain regions. To make it clear, the insert in Figure 5b shows the regions of 0% to 400%, 400% to 700%, and 700% to 1000%. The resistance of the strain sensor increased during stretching. The fitting equations of the four regions are as follows: y = 1.6804 + 7.9103x + 0.0716x^2^ (*R*^2^ = 0.9949) for 0% to 400%, y = 806.56 − 36.48x + 48.9045x^2^ (R^2^ = 0.9916) for 400% to 700%, and y = −16568.66 + 2353.3x + 13.49x^2^ (R^2^ = 0.9842) for 700% to 1000%.

To evaluate the merits of a strain sensor in practical applications, the relative resistance variation versus strain, known as the gauge factor, is evaluated; it is defined as (dR/R)/(dL/L), where R is the resistance and L is the original length of the sensor. The gauge factor represents the sensitivity of the sensor to strain. Figure 5c shows the gauge factor of the sensor under different strains. Within 400% strain, the plot follows a linear trend of y = 2.8162 + 7.1434x (Pearson’s correlation coefficient = 0.9730). The GF was determined to be 31.35 for a strain of 400%. In the 400% to 700% strain range, the plot follows a quadratic trend of y = 91.5493 − 3.4791x + 0.0716x^2^ (R^2^ = 0.9906). In the 700% to 1000% strain range, the plot follows a linear trend of y = −1653.3345 + 249.251x (R^2^ = 0.9924). As shown in Table S1, the strain sensor achieves a good balance between high sensitivity and large strain, which shows significant potential for practical applications.

A long cycling life is an important factor for practical applications. To examine the cycling stability, strains of 0 and 150% were repeatedly applied to the strain sensor over 10,000 stretching/releasing cycles; only 1000 cycles are shown in Figure 5d. The variation in the current remained unchanged over these cycles, demonstrating the good stability of the strain sensor.

### 3.4. Detection of Subtle Human Motion

**Figure 7:**
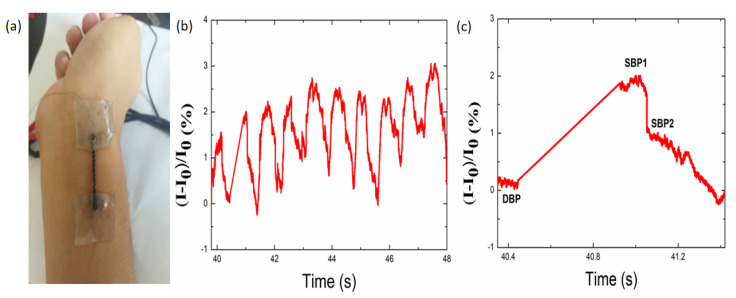
(**a**) Photograph of the TPU/graphene strain sensor with an intertwined-coil configuration assembled on the wrist of a volunteer, (**b–c**) current variation in response to the wrist pulse.

To demonstrate the potential application of the strain sensor in detecting subtle human motions, e.g., epicuticle vibration, it was mounted onto the artery of a human wrist of a healthy 180 cm tall and 20-year-old male (Figure 7a), to determine the ability of the sensor to detect the motion of the wrist. The time-dependent signals generated by the periodic wrist pulses are shown in Figure 7b. This figure shows that the detected wrist pulse detected is stable and periodic with high accuracy. Additionally, 10 obvious peaks are observed in the waveform with a 7.8 s range, indicating 77 beats per minute for the pulse rate. It should be noted that two systolic blood pressure peaks, systolic blood pressure (SBP1) and SBP2 were clearly detected, which is related to the diastolic blood, as shown in Figure 6c. The radial artery augmentation index (AIr) value, defined as ((SBP2 − DBP)/(SBP1 – diastole blood pressure (DBP))) × 100%, was calculated to be approximately 43.02%, which is in accordance with the values of people 19 years of age [64], indicating that sophisticated differences in blood pulses could be identified promptly and accurately by the strain sensor.

**Figure 8:**
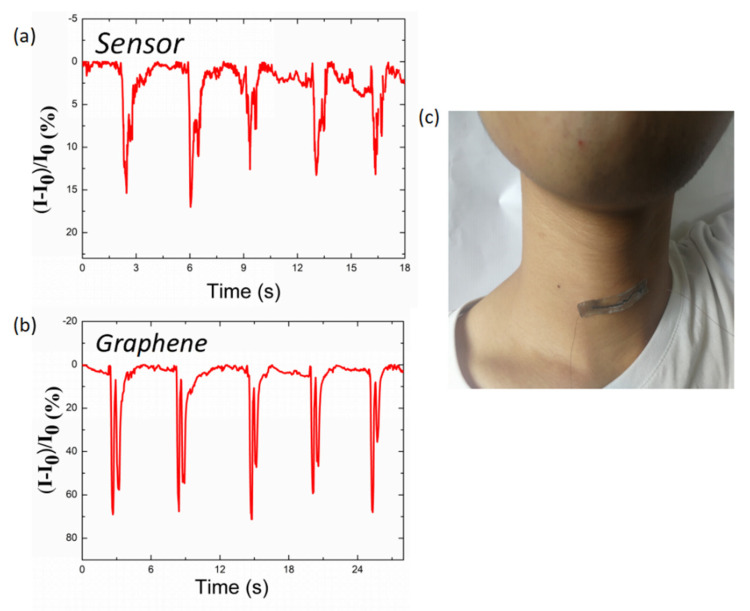
(**c**) Photograph of the TPU/graphene strain sensor with an intertwined-coil configuration attached to the throat of a volunteer. Current variation in response to speaking the words (**a**) “sensor” and (**b**) “graphene”.

Strain sensors should be able to detect physiological signals, such as phonation, by attaching the sensor onto the throat, because enabling voice actions is an indispensable part of the sensing performance. The throat muscles (Figure 8c) underwent various degrees of motion when the volunteer said different words, such as “sensor” (Figure 8a) and “graphene” (Figure 8b). Signals were generated when English letters were voiced. It is evident that each word can be distinguished easily, and the waveform and amplitudes were different for various letters. All these results demonstrated that the sensor can be used to discern different pronunciations.

### 3.5. Detection of Larger Human Motion

**Figure 9:**
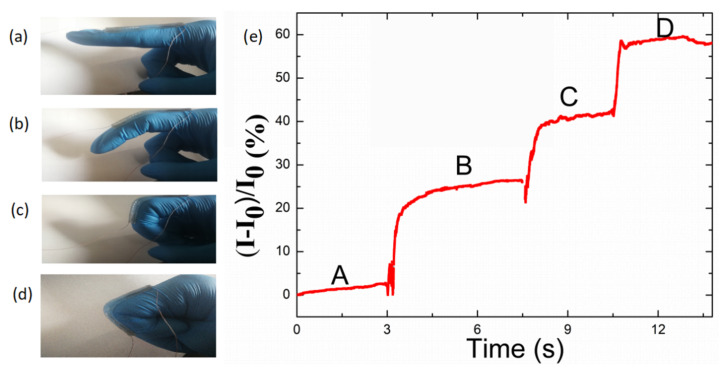
(**a**–**d**) Photograph of the TPU/graphene strain sensor with an intertwined-coil configuration attached to a volunteer’s forefinger, with different bending degrees. (**e**) Sensing performance of the TPU/graphene strain sensor with an intertwined-coil configuration for different bending degrees of the forefinger.

Apart from tiny human movements, the motion of fingers was also monitored by the sensor. In order to verify the application potential in serial detection of human motion, the sensor was attached onto a forefinger. The forefinger was bent to different angles in sequence (Figure 9b–d) from the extended state (Figure 9a), and the relative change in the current varied with the different bending angles, labeled Curve B, Curve C and Curve D, as shown in Figure 9e. The variation in the current was higher when the bending angle was larger. The reason may be that bending at a larger angle may give rise to stronger compression of the sensor.

**Figure 10:**
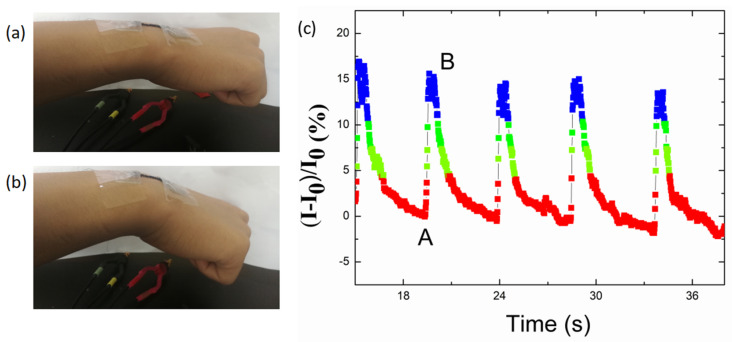
(**a**) Photograph of the TPU/graphene strain sensor with an intertwined-coil configuration attached on a volunteer’s wrist, in the initial state. (**b**) Photograph of the TPU/graphene strain sensor with an intertwined-coil configuration attached on a wrist in the bending state. (**c**) Sensing performance of the TPU/graphene strain sensor with an intertwined-coil configuration for a given bending degree of the wrist.

**Figure 11:**
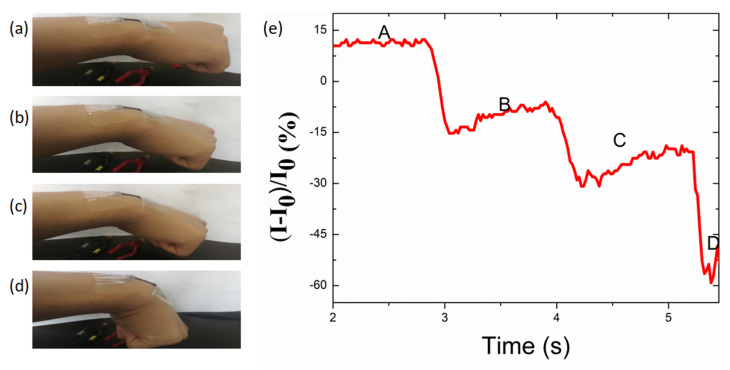
(**a**–**d**) Photograph of the TPU/graphene strain sensor with an intertwined-coil configuration attached conformally to a volunteer’s wrist, with different bending angles. (**e**) A plot of the corresponding current variation in detecting different degrees of bending of the human wrist.

In addition, the sensor was affixed on the joint of a wrist (Figure 10a,b) to obtain real-time monitoring of the motion of the wrist. Figure 10c illustrates the instant bending and unfolding motions of the wrist to a certain degree in real time, and the current could recover to its pristine state in each bending-releasing cycle. The “A” spot represents when the wrist was suddenly bent from the original state, and the shape of the curve corresponded to the bending degree of the wrist. Furthermore, the signal recovered back to the original state at the “B” spot when the wrist was entirely relaxed. Different degrees of bending of the wrist were also recorded and distinguished by the strain sensor, as shown in Figure 11. The sensor responded quickly to each degree of motion of the wrist. 

### 3.6. Detection of Pressure

**Figure 12:**
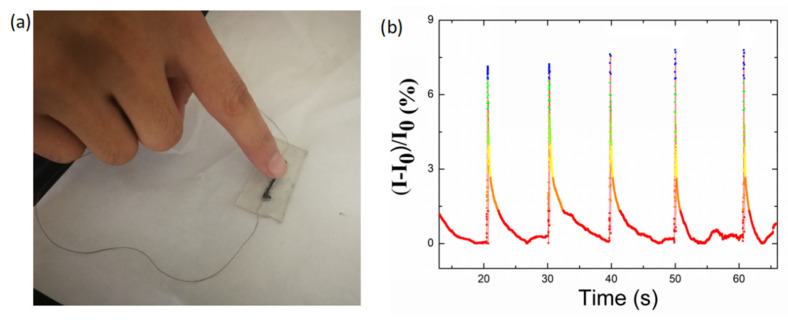
(**a**) Photograph of generating pressure by tapping of the finger. (**b**) Real-time current variation recorded by the TPU/graphene strain sensor with an intertwined-coil configuration for detection of tapping of the finger.

To further detect the sensitivity to the pressure induced by tapping of a finger, a pressure of approximately 100 Pa was generated by pressing a forefinger onto the sensor (Figure 12a). While the pressure was loaded on the sensor, the current underwent a drastic increase. The current recovered to the initial value when the pressure was withdrawn, indicating a fast sensing ability.

Specially, working temperature, magnetic fields, and humidity are the three environmental factors which would influence the sensor performance of the strain sensor. The related parameters, e.g., the temperature coefficient of resistance and the temperature coefficient of sensitivity, should all be considered in various materials and various sense types of the strain sensor, as it would be a guideline for the choice of material most suitable in different applications of our future work [65,66].

## 4. Conclusion

In summary, we presented a simple, facile assembly approach to fabricate a TPU/graphene strain sensor with a novel intertwined-coil configuration. Because of the simple procedure, the TPU/graphene strain sensor can be fabricated in a large-scale. Owing to the intertwined-coil configuration and the synergy of the two structures (nanoscale fiber gap and microscale twisting of the fibers), the new TPU/graphene strain sensor with a novel intertwined-coil configuration exhibited a stretchability of 1100%, a sensitivity of 31.35 for 400% strain, and good cycling stability. Recovery was achieved after stretching to 400%, which was higher than the limit of the range of human motion. The self-interlocking prevented the coils from uncoiling. Additionally, the configuration wrapped most of the fibers into the coils, thus avoiding their falling off of the graphene. Furthermore, the strain sensor detected the human motion induced by bending and pressure, thus demonstrating its potential as a versatile sensing tool. Additionally, it should be pointed out that mechanical dynamic analysis of various materials is necessary to be performed in our future work, for the results would provide guidelines for the choice of material for different applications [65,66]. The abovementioned stretchability as well as the moderate sensing characteristics pave the way for human motion detection and other related areas.

## Supplementary Materials

The following are available online at https://www.mdpi.com/2079-4991/10/10/1980/s1, Figure S1: (a) SEM image of TPU membrane under dip-coating of graphene for 1 time; (b) high magnification of SEM image of TPU membrane under dip-coating of graphene for 6 times; (c) SEM image of TPU membrane under dip-coating of graphene for 6 times; (d) SEM image of a coil of the TPU/graphene strain sensor in the original state; (e) high magnification SEM image of a coil of the TPU/graphene strain sensor in the original state; (f) SEM image of a coil turn into flat of the TPU/graphene strain sensor under 800 strain. Figure S2: Conductivity under various dip-coating time. Table S1: Comparison of the gauge factor and strain range for different strain sensors
