# Supplementary material for: Stretchable Strain Sensor for Human Motion Monitoring Based on an Intertwined-Coil Configuration
Source: Nanomaterials (Basel). 2020 Oct 7;10(10):1980. doi: 10.3390/nano10101980 (PMC7600075; doi:10.3390/nano10101980)
Supplement: Supplementary file 1 [file nanomaterials-10-01980-s001.pdf]

Stretchable Strain Sensor for Human Motion Monitoring Based On An Intertwined-Coil Configuration

Wei Pan [1,†], Wei Xia [1,†], Feng-Shuo Jiang [1,†], Xiao-Xiong Wang [3], Zhi-Guang Zhang [2], Xia-Gui Li [2], Peng Li [2], Yong-Chao Jiang [2], Yun-Ze Long [3,*] and Gui-Feng Yu [2,4,*]

1 College of Chemistry and Pharmaceutical Sciences, Qingdao Agricultural University, Qingdao 266109, China; tcpanwei@126.com (W.P.); xw2508717257@163.com (W.X.); nosaygiveup@126.com (F.-S.J.)

2 College of Science and Information, Qingdao Agricultural University, Qingdao 266109, China; zhangzhiguangphysics@126.com (Z.-G.Z); qdguixiali@126.com (X.-G.L.); pg.lee@163.com (P.L.); 47317810@qq.com (Y.-C.J.)

3 Collaborative Innovation Center for Nanomaterials & Devices, College of Physics, Qingdao University, Qingdao 266071, China; wangxiaoxiong69@163.com

4 State Key Laboratory of Bio-Fibers and Eco-Textiles, Qingdao University, Qingdao 266071, China

† These authors contributed equally to this work.

* Correspondence: yunze.long@qdu.edu.cn (Y.-Z.L.); xiaoyuer886as@qau.edu.cn (G.-F.Y.); Tel: +86-135 89340056 (G.-F.Y.); Fax: +86-532-86080444 (G.-F.Y.)

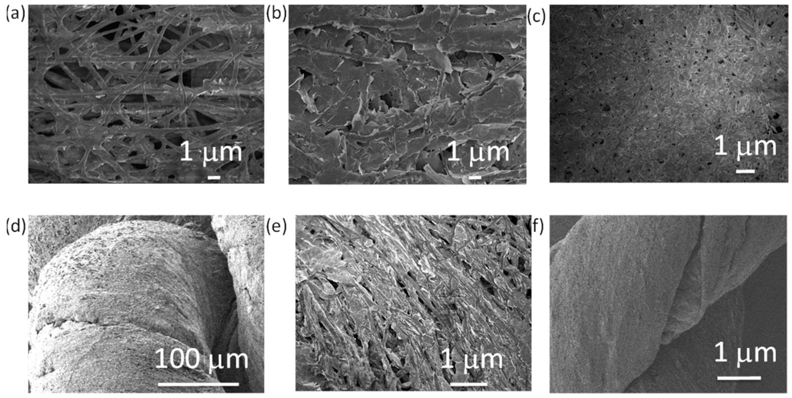

Figure S1. (a) SEM image of TPU membrane under dip-coating of graphene for 1 time; (b) high magnification of SEM image of TPU membrane under dip-coating of graphene for 6 times; (c) SEM image of TPU membrane under dip-coating of graphene for 6 times; (d) SEM image of a coil of the TPU/graphene strain sensor in the original state; (e) high magnification SEM image of a coil of the TPU/graphene strain sensor in the original state; (f) SEM image of a coil turn into flat of the TPU/graphene strain sensor under 800 strain.

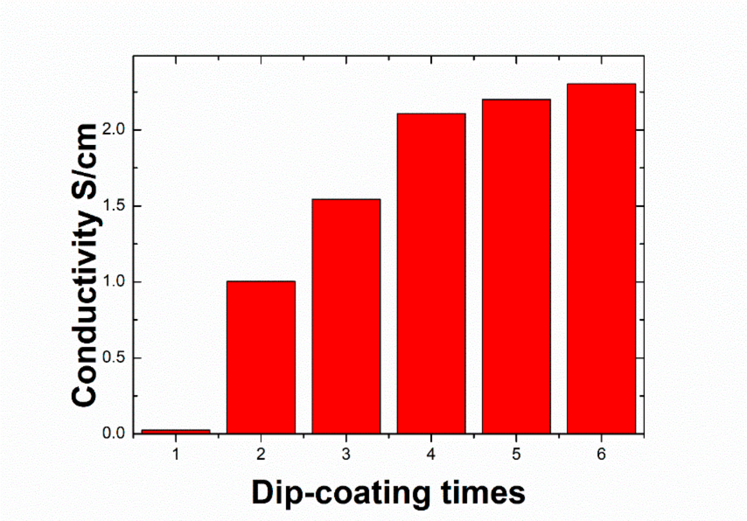

Figure S2. Conductivity under various dip-coating time.

Table S1. Comparison of the gauge factor and strain range for different strain sensors

Materials	Strain range	Gauge factor	Ref.
Graphene	8	103−106	1
Graphene	2	>150	2
Graphene	4.5	~151	3
Graphene/ionic conductor	300	25.2	4
Graphene	110	32	5
Graphene	7.1	2.4	6
Graphene	100	7.1	7
Graphene	8	500−105	8
Graphene	14.5	475	9
Graphene	110	~	10
Graphene	1.5	150	11
TPU/Graphene	1100	31.35	This work

1. Hempel, M.; Nezich, D.; Kong, J.; Hofmann, M., A novel class of strain gauges based on layered percolative films of 2D materials. *Nano letters* **2012,** *12* (11), 5714-5718.
2. Li, X.; Zhang, R.; Yu, W.; Wang, K.; Wei, J.; Wu, D.; Cao, A.; Li, Z.; Cheng, Y.; Zheng, Q., Stretchable and highly sensitive graphene-on-polymer strain sensors Sci. *Rep* **2012,** *2*, 870.
3. Fu, X.-W.; Liao, Z.-M.; Zhou, J.-X.; Zhou, Y.-B.; Wu, H.-C.; Zhang, R.; Jing, G.; Xu, J.; Wu, X.;

Guo, W., Strain dependent resistance in chemical vapor deposition grown graphene. *Applied Physics Letters* **2011,** *99* (21), 213107.
4. Lin, Y.; Liu, S.; Chen, S.; Wei, Y.; Dong, X.; Liu, L., A highly stretchable and sensitive strain sensor based on graphene–elastomer composites with a novel double-interconnected network. *Journal of Materials Chemistry C* **2016,** *4* (26), 6345-6352.
5. Wu, S.; Peng, S.; Han, Z. J.; Zhu, H.; Wang, C. H., Ultrasensitive and stretchable strain sensors based on mazelike vertical graphene network. *ACS applied materials & interfaces* **2018,** *10* (42), 36312-36322.
6. Bae, S.-H.; Lee, Y.; Sharma, B. K.; Lee, H.-J.; Kim, J.-H.; Ahn, J.-H., Graphene-based transparent strain sensor. *Carbon* **2013,** *51*, 236-242.
7. Yan, C.; Wang, J.; Kang, W.; Cui, M.; Wang, X.; Foo, C. Y.; Chee, K. J.; Lee, P. S., Highly stretchable piezoresistive graphene–nanocellulose nanopaper for strain sensors. *Advanced materials* **2014,** *26* (13), 2022-2027.
8. Wang, Y.; Wang, L.; Yang, T.; Li, X.; Zang, X.; Zhu, M.; Wang, K.; Wu, D.; Zhu, H., Wearable and highly sensitive graphene strain sensors for human motion monitoring. *Advanced Functional Materials* **2014,** *24* (29), 4666-4670.
9. Yang, Z.; Wang, D.-Y.; Pang, Y.; Li, Y.-X.; Wang, Q.; Zhang, T.-Y.; Wang, J.-B.; Liu, X.; Yang, Y.-Y.; Jian, J.-M., Simultaneously detecting subtle and intensive human motions based on a silver nanoparticles bridged graphene strain sensor. *ACS applied materials & interfaces* **2018,** *10* (4), 3948-3954.
10. Yuan, W.; Yang, J.; Yang, K.; Peng, H.; Yin, F., High-performance and multifunctional skinlike strain sensors based on graphene/springlike mesh network. *ACS applied materials & interfaces* **2018,** *10* (23), 19906-19913.
11. Jiang, L.; Fan, Z., Design of advanced porous graphene materials: from graphene nanomesh to 3D architectures. *Nanoscale* **2014,** *6* (4), 1922-1945.
